# Is it time to abandon care planning in mental health services? A qualitative study exploring the views of professionals, service users and carers

**DOI:** 10.1111/hex.12650

**Published:** 2017-11-16

**Authors:** Helen L. Brooks, Karina Lovell, Penny Bee, Caroline Sanders, Anne Rogers

**Affiliations:** ^1^ Department of Psychological Sciences Institute of Psychology Health and Society University of Liverpool Liverpool UK; ^2^ Mental Health Research Group Division of Nursing, Midwifery and Social Work Faculty of Biology, Medicine and Health School of Health Sciences Manchester Academic Health Science Centre University of Manchester Manchester UK; ^3^ NIHR School for Primary Care Research Division of Population Health Faculty of Biology, Medicine and Health School of Health Sciences Manchester Academic Health Science Centre University of Manchester Manchester UK; ^4^ Faculty of Health Sciences NIHR CLAHRC Wessex University of Southampton Southampton UK

**Keywords:** care planning, experiences of care, mental health, qualitative, user/carer involvement

## Abstract

**Background:**

It has been established that mental health‐care planning does not adequately respond to the needs of those accessing services. Understanding the reasons for this and identifying whose needs care plans serve requires an exploration of the perspectives of service users, carers and professionals within the wider organizational context.

**Objective:**

To explore the current operationalization of care planning and perceptions of its function within mental health services from the perspectives of multiple stakeholders.

**Settings and participants:**

Participants included 21 mental health professionals, 29 service users and 4 carers from seven Mental Health Trusts in England. All participants had experience of care planning processes within secondary mental health‐care services.

**Methods:**

Fifty‐four semi‐structured interviews were conducted with participants and analysed utilizing a qualitative framework approach.

**Findings:**

Care plans and care planning were characterized by a failure to meet the complexity of mental health needs, and care planning processes were seen to prioritize organizational agendas and risk prevention which distanced care planning from the everyday lives of service users.

**Discussion and conclusions:**

Care planning is recognized, embedded and well established in the practices of mental health professionals and service users. However, it is considered too superficial and mainly irrelevant to users for managing mental health in their everyday lives. Those responsible for the planning and delivery of mental health services should consider ways to increase the relevance of care planning to the everyday lives of service users including separating risk from holistic needs assessment, using support aids and utilizing a peer workforce in this regard.

## 1 INTRODUCTION

The need to incorporate principles of service user and carer involvement in the planning and delivery of care is predicated on the idea that this can lead to positive outcomes for health‐care systems and their users.^1^, ^2^, ^3^, ^4^, ^5^ Benefits include increased engagement with and a more positive experience of care,^5^, ^6^, ^7^ improved service quality,^8^ reduced rates of enforced treatment and readmission^9^ and reduced stigma and social isolation.^7^, ^8^, ^9^

Care planning is an area of identified contemporary practice in which user involvement principles are suboptimal but can, in principle, be realized in practice.^5^, ^6^, ^7^, ^8^, ^9^, ^10^, ^11^, ^12^ Mental health‐care planning has been defined as the process through which services in relation to an individual's care are “assessed, planned, co‐ordinated and reviewed”.^13^ In the United Kingdom, current NICE guidelines state that “people using mental health services [should] develop a care plan with mental health and social care professionals, and [be] given a copy with an agreed date to review it.”.^14^ Despite the embedding of engagement and involvement principles in policy and practice ideologies, current evidence suggests that service users and carers feel unsupported by and distanced from care planning processes and consistently report a desire for greater involvement.^8^, ^15^, ^16^ This applies across a variety of service settings and professional roles.^12^, ^17^ Barriers to service user and carer involvement in mental health services include a lack of a shared definition of involvement,^5^ the administrative burden of care plans,^12^ poor information exchange,^5^ limited opportunities for involvement,^5^, ^15^ ritualized practices^5^ concerns about confidentiality^15^ and inhibitions based on historical use and contemporary associations of potential coercion within mental health services.^18^, ^19^, ^20^

Current care planning practices should not be considered in isolation from the wider contextual agenda which shapes systems and processes. For example, care planning forms part of a number of elements that are subjected to quality assurance. The use of such quality indicators is driven by demands for transparency and accountability with organizations placing emphasis on the need for measurement and evaluation of performance.^21^, ^22^ This demand for evidence of performance management in mental health services has been accentuated by the introduction of the Joint Commissioning Panel for Mental Health and local commissioning groups.^23^ The increased significance of such indicators within health services has produced unintended consequences.^24^, ^25^ In the context of primary care, this has included the tendency to focus on isolated aspects of care, which may lead to poor care for service users.^24^ Other negative consequences include overtreatment and “tunnel vision” whereby professionals focus on problem areas inherent in quality indicators to the detriment of other areas of practice.^24^, ^25^ Thus, the way in which needs assessments and care planning are framed and executed as part of quality assurance, improvement and governance agendas is relevant to understanding the perception of their primary use as a means of organizing and planning care in a way which meets patient need. For example, we know that service users and carers themselves attach priority value to relational aspects of care planning compared to professionals who focus instead on service‐led outcomes.^5^, ^26^, ^27^

Current quality indicators associated with the processes of mental health‐care planning are gathered from a variety of sources. They include experience surveys and feedback from service users relating to the number of users who report having a jointly agreed care plan, the proportion reporting being given a care plan and the number who report being given an agreed date to review their care plan.^14^

This study sought to understand service user and carer involvement in care planning by exploring the current use of care planning processes within mental health services and the impact of contextual factors on the quality of care planning provided. Perspectives of multiple stakeholders including service users, carers and professionals were elicited to this end.

### 1.1 Aim

To explore the current operationalization of care planning within mental health services from the perspectives of multiple stakeholders.

## 2 METHODS

### 2.1 Study design

A qualitative study utilizing semi‐structured interviews formed part of a wider programme of research designed to improve service user and carer involvement in mental health‐care planning. The presentation of methods and results is informed by the Consolidated Guidelines for the Reporting of Qualitative Data.^28^ Ethical approval was obtained from the National Research Ethics Committee North West‐Lancaster [14/NW/0297].

### 2.2 Participants

Inclusion criteria included mental health service users, carers or professionals from community secondary mental health‐care services already recruited to a randomized controlled trial testing the effectiveness of a training package for professionals to improve service user and carer involvement in care planning. Service user participants from the trial were invited to take part in this study through a written invitation. Interested participants returned a consent‐to‐contact form to the research team who then contacted them directly via email or phone. Staff members who were interested in taking part in the study responded to an email invitation and service users were asked to identify relevant carers for inclusion in the study. Additional criteria included being aged 18 or over. The study utilized purposive sampling to ensure adequate representation across gender and geographical area.

In total, 87 participants (31 professionals, 47 service users and 9 carers) expressed an initial interest in taking part. HB contacted participants to ascertain eligibility and to arrange an interview (see Table** 1 for further details of study participants). Fifty‐four participants (21 professionals, 29 service users and 4 carers) from seven Mental Health Trusts in England provided informed consent to participate (Table** 1). Reasons for non‐participation included non‐response and participants no longer wishing to take part in the study because of, for example, illness, changes in circumstances or a lack of time.

**Table 1:** Demographic information

Service users	
Male	13
Female	16
Manchester Mental Health and Social Care Trust	12
Nottinghamshire Healthcare	5
South west Yorkshire Partnership NHS Trust	3
Leicestershire NHS Trust	7
Greater Manchester West NHS Trust	2
Total	29
Carers	
Male	2
Female	2
Manchester Mental Health and Social Care Trust	1
Nottinghamshire Healthcare	1
South west Yorkshire Partnership NHS Trust	1
Leicestershire NHS Trust	1
Total	4
Professionals	
Male	3
Female	18
Manchester Mental Health and Social Care Trust	9
South west Yorkshire Partnership NHS Trust	2
Pennine Care NHS Trust	1
Leeds and York Partnership NHS Trust	9
Total	21

Participants were provided with an information sheet and given the opportunity to ask questions about participation prior to completing consent forms. Those undertaking the interview over the phone (professionals = 10, service users = 4, carers = 3) returned consent forms prior to data collection. Participants took part in three interviews over 12 months. This study reports on findings from the baseline interviews only.

### 2.3 Data collection

Self‐reported demographic information was collected prior to data collection in order to contextualize the data presented. Interviews were conducted by HB at a convenient time and place for participants and the option to complete the interview via the telephone was offered. Face‐to‐face interviews were carried out on trust or university premises, in participants’ homes or at another convenient community location. Two female service user participants had a significant other present during the interview at their request. These individuals were present during interviews but were not participants and did not contribute to interview data.

Digitally recorded semi‐structured interviews lasting between 20 and 70 minutes were undertaken between August 2014 and January 2016. Interviews were semi‐structured in nature to allow for the introduction of new themes by study participants. Interview schedules informed by existing literature^5^, ^8^, ^12^, ^15^, ^16^, ^17^, ^18^, ^19^, ^20^ explored experiences of contemporary care planning within mental health services and organizational processes and systems related to care planning (see Appendix** 1). Data collection stopped when consensus amongst the research team was reached that data saturation had occurred.

### 2.4 Data analysis

Digital recordings were transcribed verbatim by an independent and experienced transcription company before being anonymized and allocated to a member of the study team (HB or AR). The first 10 transcripts were coded independently by HB and AR who familiarized themselves with the data before starting the inductive coding process.^29^ The authors then met to discuss emerging themes and to produce a provisional framework.^30^ The remaining transcripts were coded by HB using NVivo to organize the data. Over the course of this coding, all data relevant to a category were identified and examined using constant comparison where new categories were added to the framework to reflect as many nuances within the data as possible.^31^ Emergent findings were presented to the wider study Service User and Carer Advisory Group (SUCAG) to ensure interpretations of data were grounded in the experience of mental health services. The study team discussed revisions arising during the analysis process before agreeing a final framework to present the results, which was considered representative of the entire data set. An excel document detailing basic demographic information for each study participant was used to contextualize and organize data.

HB is a Lecturer and Health Service Researcher, PB a Reader in Mental Health, KL is a Professor in Mental Health, CS is a Senior Lecturer in Medical Sociology, and AR is a Professor of Health Systems Implementation. Researchers had neither prior relationships nor current therapeutic relationship with participants. The starting point of the study was one informed by the value of involving service users and carers in the design and delivery of mental health care.^32^ All interviews were undertaken by HB, a female postdoctoral Research Fellow with significant qualitative research experience.

### 2.5 Findings

Despite being aware of the care planning process, the majority of service users and carers included in this study had neither seen their care plan nor been involved in its development. The minority who did report having seen a care plan did not consider the plan to be useful to the management of their mental health conditions or their future recovery. There was a general consensus amongst all participant groups that care plans were of most relevance to professionals and mostly inconsequential to the everyday lives of service users.

It [the care plan] was a case take your medication, watch TV and don't, let anybody jump on you and don't jump on anybody else.

(SUIV1020, service user, female)

I just think it [the care plan] becomes, for some people, it's part of that nursing process that, kind of, isn't anything to do with them [service users].

(PROFIV1019, professional, female)

One of the main reasons identified for this irrelevance was the lack of the required multidisciplinary working to adequately address managing the complexity of mental health conditions or to take on board holistic or broader contextual and environmental influences impacting on a person's ability to manage their condition. Rather, care planning processes prioritized organizational and risk agendas which further distanced and alienated the process of care planning from the everyday lives of service users. There were no discernable differences in this regard from the data collected from service users and carers.

### 2.6 Managing complexity—Multidisciplinary working within mental health services

Participants felt that the process of care planning subjected complicated human processes (eg the experience of serious mental illness) to overly simplistic frameworks which were not sensitive to the vagaries of living with a long‐term mental health problem. At times service users were experiencing thoughts, emotions or medication effects which made it difficult to engage in care planning discussions.

It's tricky because everything moves and fluctuates every time you see someone. So that care plan is relevant for that week or that month that you did it but you do review them six monthly or six monthly to a year, depending on the person, and it can change quite frequently.

(PROFIV1014, professional, female)

Sometimes I've been so ill I just, it's been enough just to get through the day and I, I didn't really want a care plan. And I didn't want to be involved in it. Because I was so ill I just, I was just surviving really.

(SUIV1001, service user, male)

Additionally, the care planning process did not fully take into account the implicit coercive elements of mental health services. Service users who were currently or had previously been subject to service provision against their will through detention under the Mental Health Act saw little value in engaging in the delivery of management plans they did not want nor had control over in the first place and this was acknowledged by mental health professionals.

I kind of feel like he's going to act on it anyway, because I suppose in past experiences that's what professionals have done, they've took that control out of my hands and they've just done something because they feel like it's in my best interest.

(SUIV1028, service user, female)

Some people do not want to engage in that. It's not something that they feel they have any affinity to and they just do not want that. They see it as an invasion, they don't want mental health services in their life.

(PROFIV1010, professional, female)

Care planning has traditionally been predicated on a multidisciplinary approach to service delivery with a stated objective of ensuring that the holistic needs of services users are met. However, professionals felt that the fragmentation of existing services and limited resources made this difficult to realize in practice. Service users also talked about the problems of a system that failed to adopt an holistic approach to planning which reflected their real‐life priorities and ability to leverage resources which could be of assistance.

Obviously if you've got a patient who's admitted to a ward and you're needing to go to a CPA review they'll normally dictate it around the consultant's availability and if you're lucky you'll get an invite or be told about it and if you are, they're not very flexible with timing. So as a care co‐ordinator as a team we've often about 50 or more service users on caseloads. It's really impractical to only be given a week's notice and it's this day, this time, that's it. It's really, really difficult for the team.

(PROFIV1015, professional, female)

You get pockets of care, pockets of support you, which you have to perhaps develop for yourself. Like I had a worker to try and help me with my housing but that wasn't joined up to say, my review appointments with the psychiatrist, that wasn't even spoken about.

(SUIV1020, service user, female)

Professionals acknowledged that care plans were mostly written by a care coordinator located in a community context but in isolation from other professionals and other parts of the health system. This lack of a multidisciplinary approach was compounded by IT systems, which were perceived to inhibit intraprofessional communication. Professionals felt that IT systems restricted service user involvement in the care planning process; documents were described as “utilitarian” (PROFIV1001, professional, male) and were not considered user‐friendly. Additionally, systems did not lend themselves to remote working.

There are hindrances to the process in that we haven't got remote working. It would be much better if we could actually take a laptop out or a tablet or something with our system on it.

(PROFIV1003, professional, female)

Circumventing such systems often necessitated localized, creative working from professionals and involved undertaking activities in addition to mandatory care planning.

If you've got a client with say autism or Asperger's which is a good example for them to communicate to you what it is they're feeling, what it is they're going through some clients the best way is not on a piece of paper and you writing it down. So something that you might do is get a blank piece of paper and do a collage so they can explain through pictures pulled from a magazine or pictures off the internet or symbols or signs how it is that they're feeling and where it is they want to be.

(PROFIV1018, professional, female)

Participants coalesced in their views that limited resources both within and outside health services impacted on multidisciplinary and holistic approaches to care planning and meant there was often little tangible benefit to service users and carers of engaging with the care planning process. Needs may be identified but participants spoke consistently of there being no resources available to address those needs.

There are times when you might say something to your CPN and [pause] you see almost a smile come on their lips as they say, I'm sorry, we can't do that. And you know perfectly well the bean counters back at the Trust, the CPNs just can't manage it.

(SUIV1009, service user, male)

I don't think there's the funding to do the little extra bits that would make things much easier all round for both the patients and the carers really.

(CARER1004, carer, female)

### 2.7 Feeding the machine—care planning as a process designed to serve the system

There was a general consensus amongst participants that another reason care plans were divorced from service user needs was because care planning processes currently served the mental health system rather than those who accessed care. It was described as a “*bureaucratic process that's imposed on them [service users] to [enable them] to get help (PROFIV1001, professional, male).”* Participants felt that care plans were currently being used as a tool for professional communication, as a way of monitoring professional behaviour and as an audit trail if things went wrong (eg in coroner's court).

I think it's to give them a sense of purpose to say that they're actually going to do something for you to make you better, but to tell you the truth, I think it was just to lay it down to say, yeah, we're going to do X Y and Z, and stuff like that, and all it amounted to was, like, coming around to visit you, you know.

(SUIV1025, service user, male)

Care planning was seen as something that “had to be done” to serve bureaucratic expediency rather than any dynamic user centred needs.

The consensus that I've got from the team at the moment, and I'll be honest, is that it's just another document that they have to go through to tick the boxes.

(PROFIV1015, professional, female)

I think they [professionals] see it as something that has to be done. And they, sort of, get in the way a little bit… And what time they spend on care plans they can't spend talking to me, as it were.

(SUIV1002, service user, female)

As such, they were written in professional language in a way that served organizational agendas. For example, professionals reported how for service users in supported accommodation care plans had to clearly state a service user's needs in order to leverage funding from housing provider organizations.

It's a shame, I mean really for all those people you could ideally do with a separate document, you know, but again it's like we already have so much bureaucracy. We have to highlightlots of kind of risky things on it that the client might not maybe be experiencing at the time, but we have to put them in in order to either get the money or if the person becomes ill.

(PROFIV1002, professional, female)

Professionals reported constant pressure from the imperative of organizational targets that impacted directly on the quality of developing care plans and subsequent patient care. Such an inflexible system was seen to generate standardization, which left little room for diversification and creative solutions.

We've got pressurised timescales to get the CPA planning in place for…for government targets. The pressure actually pushes us towards rushing it really…rather than taking our time in the care planning process.

(PROFIV1003, professional, female)

All participants described care plans as lengthy documents requiring substantial and recurrent assessments directed at achieving organizational targets. Service users and carers placed more value on patient‐centred discussions undertaken as conversations, which eschewed formal assessment. Service users talked about how connection and understanding were of value to them rather than the care planning process, which could be viewed as a direct barrier to this form of relationship.

It being on paper isn't a priority for them. You coming to visit and having those conversations are, you know, doing the other kind of work that you do…is maybe more of a priority.

(PROFIV1011, professional, female)

The majority of targets related to care planning identified by professionals were presented as a set of binary outcomes such as “is the care plan in date?” rather than any indication of substantive content (eg the quality and utility of information contained within the care plan). Participants described how these targets provided sufficient evidence for managers about performance levels within care teams. However, this often did not reflect what happened in practice. Care coordinators reported copying across previous care plans without reviewing them to make the system believe that care plans were in date. In some cases, this approach was being actively encouraged by managers to relieve system pressures.

We've been told by a manager that yes we know these are long and laborious but just put a start date and an end date on them and fill them in later because then it will show on the computer system that you've done a care plan and get it done on the day that you meet the person. And that not only is frustrating but it's morally wrong, it's just ticking a box and it's not respectful of the person and their difficulties.

(PROFIV1018, professional, female)

Service users and carers were unaware of individual pressures and targets and instead referred to a more general sense of bureaucratic pressure. A small number of service users described “going along” with bureaucratic imperatives to avoid potential sanctions, to make their lives easier, or to “help” professionals meet organizational targets without the process having any tangible benefit to them.

I just agree with what they say really, coz it makes it easier that way. But underneath I'm just don't really care you know.

(SUIV1001, service user, male)

### 2.8 Risk prioritization and the perceived vulnerability of the role of care coordinator

Both service users and professionals described a predominant focus on risk management within current care planning processes.

The risk policy is around reviewing risk whenever there's a change in risk, so we have I think it's a twelve…is it a six or twelve page risk assessment. There's one major risk assessment but then we have a risk review which is like a tick sheet, so if somebody, um, like your CPA review, you review the risk and do a risk follow up, or…if something happens between CPAs, that's significant, so if somebody's risk or frequency or severity of self‐harming went up or they're expressing threats to harm someone, outside of CPA reviews, you'd be expected to review that.

(PROFIV1001, professional, male)

Professionals described the limited time they often had to spend with service users which meant there was a focus during appointments on agendas prioritized by the organization such as risk assessments rather than working towards longer term recovery goals which further distanced service activities from the everyday lives of service users.

I mean, we just sit around and talk and then he goes, oh, I've got to go now, and you go, alright, I'll see you in two weeks or you phone him up and say, well, I won't be here in two weeks because, you know, you're not going to kill yourself, no, I'm just going out, you know, so… [] it's containing that rather than moving forward. [risk]

(SUIV1025, service user, male)

Your caseload is looked at and if you've got those documents in place then they're not…that case is flagged up. However, if you don't have the care plan or you don't have a risk assessment or whatever, in place, then you're then asked why, why isn't that happening?

(PROFIV1017, professional, female)

Some service users appreciated why professionals focussed on risk but this could bring back unhappy memories related to periods of acute illness.

I think they have to make sure that we won't be taking an overdose, so I think they've got to be careful

(SUIV1001, service user, male)

Do you talk about risk a lot within your care planning meetings?

Yes. They have to do a risk assessment anyway. It's a bit scary really ‘cause it makes me realise how off the rails I can get.

(SUIV1002, service user, female)

Professionals also described how this risk focus was related to the role of care coordinators within community mental health teams. Care coordinators were seen to hold substantial responsibility for service users on their caseload whilst concomitantly working in isolation from other members of the team. This perceived isolation led to a sense of vulnerability being attached to the role of care coordinator if things went wrong which resulted in increased focus on managing potential risk.

It tends to be the admin that isn't done, and I think the concern is that if something happens to somebody and you haven't done…if there's no care plan or obviously a risk assessment is not up to date, or if you haven't had time to do something that you should have done, that you're going to end up in coroner's court—that's probably the main thing. Or that something happens to somebody and there's more that you could have done, and you'd have to live with that.

(PROFIV1016, professional, female)

This perceived isolation was compared to staff working within hospitals where it was imagined that responsibility was more likely to be dispersed between members of the interdisciplinary team. Participants described how staff in hospital settings could hand over work to colleagues once they left the ward. Care coordinators in the community on the other hand did not appear to have the option to hand over workload and often reported taking work concerns home with them and worrying about what would happen to individuals on their caseload. Expressed concerns about stress and burnout amongst professionals as a result of this burden were frequent.

I mean we can't carry on as we are because we're going to end up with people going off, off sick, and things like that. Everybody is…well, like, my colleagues, we talk about it, people are feeling it, people are not sleeping and people are worrying, and it's really getting into your home life at the moment.

(PROFIV1016, professional, female)

## 3 DISCUSSION

The question posited at the outset of this manuscript was ‘is it was time to abandon care planning in mental health services?’ The study answered this by exploring service user involvement in care planning within the wider context of the mental health‐care system from the perspectives of multiple stakeholders. The study did not seek to reach consensus but instead to position multiple versions of the experience of care planning alongside each other. A key finding of this study relates to the lack of alignment of care planning activities to the everyday lives of service users. In this respect, care planning was seen to be fulfilling one organizational goal seemingly at the expense of delivering the primary stated purpose of care planning. Rather than abandoning care planning in mental health services, various ways in which services could adapt processes to increase the relevance of care plans to the everyday lives of service users are considered.

The definitions of care planning purported by health organizations^14^ describe the inclusion of patients’ experience in the planning and management of mental health. However, the focus on current quality indicators related to care planning fail to harness this experiential component and concentrate instead on binary, quantitative outcomes (eg does the patient have a copy of their care plan?) and risk assessments. It has previously been demonstrated that whilst patients see a benefit in discussing risk, this is understood in terms of a professional priority that may lead to loss of liberty.^33^ The current study adds to this by highlighting that the focus on quality indicators along with the inclusion of organizational risk assessments within current care planning structures also detracts from meeting the expectations or expressed needs of service users.

“Measurement fixation” is an unintended consequence of systems designed to measure performance of professionals.^24^ There is increasing recognition of the need to consider better ways of capturing patient and care experiences in a more meaningful way in specific care contexts.^22^ This study adds to existing literature through demonstrating that the unintended outcomes of quality indicators are manifest within mental health‐care planning systems with the consequence that the intended focus of care plans, responding to needs in a holistic and patient‐centred way, is thwarted and preference is given instead to feeding organizational imperatives for measuring performance. This consequence is reinforced by the original impetus of the care planning approach as a mechanism to feedback to commissioners in relation to activity and risk management.^19^

Organizational processes reported in the current study focussed on paternalistic, formulaic approaches to risk, serving organizational accountability agendas related to the origination of care planning as a result of concerns about safety and fragmented community care.^12^ The findings support the recent literature demonstrating that actuarial risk assessments can be used by professionals to manage uncertainty in a manner that distances service users from potential solutions.^33^, ^34^, ^35^ To better integrate care plans with people's everyday lives, risk management should be separated from holistic needs elicitation. The latter could be elicited through tailored dialogue between service users and professionals with the former developed separately through formulaic and less engaging processes.

In order to reorient care plans to the everyday lives of mental health service users, planning discussions could be supported by evidence‐based tools designed to open up opportunities for accessing resources to help manage a condition and meet need.^36^ Given their fit with an individual's real‐world environment and everyday management of mental health conditions, such interventions may address the perceived invisibility of care plans in relation to meeting the needs of service users. Using a peer workforce to complement that provided by health professionals may be useful to take care planning in a different, more user‐focused direction away from the organizational constraints, paternalistic culture and clinical norms of surveillance and control associated with statutory services.^36^, ^37^, ^38^

The study gains its strengths from the insightful data gathered from the in‐depth nature of the methods employed and the ability to compare data across multiple stakeholder groups. However, the data reflect the experiences of stakeholders at one point in time and do not purport to reflect the experiences of all mental health service users, carers and professionals. Generally, the negative experiences recalled by participants speak to a wider discontent with mental health services, which has been documented previously,^5^, ^15^, ^16^ but the focus on the current use of care planning within services has illuminated some of the potential structural factors underlying this discontent and has identified potential areas for intervention. Whilst carer participants’ views coalesced with service user views and their concerns supported those reported previously,^15^ only four carer participants took part in the study so their views may be under‐represented in the data presented.

## 4 CONCLUSION

Service user involvement in care planning represents a key focus of global mental health policy. The present study found that the current operationalization and utilisation of care planning represent significant barriers to this process. Those responsible for the planning and delivery of mental health services should consider ways to increase the relevance of care planning to the everyday lives of service users including separating risk from holistic needs assessment, using support aids and utilizing a peer workforce in this regard.

## COMPETING INTERESTS

The authors declare no competing interests.

## APPENDIX 1 INTERVIEW SCHEDULES

### PROFESSIONAL

- Can you please tell me a little bit about your role in care planning to date?
- What is the current formulation of care planning expected to do?
  ○ Does it achieve these aims?
  ○ What actually happens in meetings?
  ○ How do the health professionals effectively engage participants and arrive at a plan?
  ○ Who is involved in these meetings?
  ○ How much does it influence the nature of management/care?
  ○ Query policy, targets and general profile of CP?
  ○ Does it look at or tap into service user networks/resources?
  ○ Examples of good care planning?
  ○ Examples of bad care planning?
  ○ Any alternatives?
- How is care planning understood by other staff and service users across services?
- What are staff and professionals attitudes towards current care planning?
- What elements currently missing in context to make it work properly for professionals?
- What would make the biggest difference to improving mental health services currently?

### SERVICE USER/CARER

- Who or what help you manage your mental health condition on a day‐to‐day basis?
  ○ Complete network diagram
  ○ Prompt: range of network members
- Can you please tell me a little bit about your experience care planning to date?
- What do you think care planning is currently expected to do?
  ○ Do you know how care planning works within services and how do know (where has information come from? (Probe shared definition and understanding amongst service users and professionals)
  ○ Can you describe the format of a meeting? (Make this in general first and then ask them to focus on one key one or the last one. Get them to identify it before asking in depth about it.)
  ○ Who is involved in these meetings? (Again typically and last one attended).
  ○ How much does what goes on in meetings translate into what you experience on a day‐to‐day basis?
  ○ How often do you think about what you discussed in the care planning meeting and can you give examples of something it has effected (Probe specific examples and how well it works)
  ○ In your experience have you been asked about how and what you manage with? Do professionals ask about different areas of your life (e.g. your pets your relationships what you can do to keep yourself occupied as well as medicine etc.
  ○ Examples of good care planning)?
  ○ Examples of bad care planning (These can be own experiences or people you know or have heard of or something you have read)?
  ○ Are there any alternatives to care planning that you know of or have had experience of?
- What are staff attitudes towards current care planning?
- Are there any differences between inpatient and community care?
- What elements currently missing in context to make it work properly for you?
- What would make the biggest difference to improving mental health services currently?

## References

[1] Department of Health . Refocusing the Care Programme Approach. Policy and Positive Practice Guidance. London: Department of Health; 2008.

[2] Commonwealth of Australia . Fourth National Mental Health Plan: An Agenda for Collaborative Government Action in Mental Health 2009‐2014. Canberra, ACT: Commonwealth of Australia; 2009.

[3] World Health Organisation . Draft mental health action plan: an overview. 2012 http://www.who.int/mental_health/mhgap/2_11_2012_Saxena.pdf. Accessed May 31, 2017.

[4] Simpson EL , House AO . User and carer involvement in mental health services: from rhetoric to science. Br J Psychiatry. 2003;183:89‐91. 12893657 10.1192/bjp.183.2.89

[5] Bee P , Price O , Baker J , Lovell K . A systematic synthesis of barriers and facilitators to user‐led care planning. Br J Psychiatry. 2015;207:104‐114. 26243762 10.1192/bjp.bp.114.152447 PMC4523927

[6] Crawford MJ , Aldridge T , Bhui K , et al. User involvement in the planning and delivery of mental health services: a cross‐sectional survey of service users and providers. Acta Psychiatr Scand. 2003;107:410‐414. 12752016 10.1034/j.1600-0447.2003.00049.x

[7] Thornicroft G , Tansella M . Growing recognition of the importance of service user involvement in mental health service planning and evaluation. Epidemiol Psichiatr Soc. 2005;14:1‐3. 10.1017/s1121189x00001858 15792287

[8] Richards DA , Lankshear AJ , Fletcher J , et al. Developing a UK protocol for collaborative care: a qualitative study. Gen Hosp Psychiatry. 2006;28:296‐305. 16814628 10.1016/j.genhosppsych.2006.03.005

[9] Henderson H , Flood C , Leese M , Thornicroft G , Sutherby K , Szmuckler G . Effect of joint crisis plans on use of compulsory treatment in psychiatry: single blind randomised controlled trial. BMJ. 2004;329:136. 15240438 10.1136/bmj.38155.585046.63 PMC478218

[10] Gately C , Rogers A , Sanders C . Re‐thinking the relationship between long‐term condition self‐management education and the utilisation of health services. Soc Sci Med. 2007;65:934‐945. 17521790 10.1016/j.socscimed.2007.04.018

[11] Rogers A , Day J , Randall F , Bentall RP . Patients’ understanding and participation in a trial designed to improve the management of antipsychotic medication: a qualitative study. Soc Psychiatry Psychiatr Epidemiol. 2003;38:720‐727. 14689177 10.1007/s00127-003-0693-5

[12] Simpson A , Hannigan B , Coffey M , et al. Recovery‐focused care planning and coordination in England and Wales: a cross national mixed methods comparative case study. BMC Psychiatry. 2016;16:147. 27184888 10.1186/s12888-016-0858-x PMC4868048

[13] NHS Choices . Care programme approach. 2015 http://www.nhs.uk/conditions/social-care-and-support-guide/pages/care-programme-approach.aspx. Accessed June 31, 2017.

[14] NICE . Service user experience in adult mental health services. Quality standard [QS14]. 2011 https://www.nice.org.uk/guidance/qs14/chapter/quality-statement-8-care-planning. Accessed May 31, 2017.

[15] Cree L , Brooks H , Berzins K , Fraser C , Lovell K , Bee P . Carers’ experiences of involvement in care planning: a qualitative exploration of the facilitators and barriers to engagement with mental health services. BMC Psychiatry. 2015;15:208. 26319602 10.1186/s12888-015-0590-y PMC4553006

[16] Grundy A , Bee P , Meade O , et al. Bringing meaning to user involvement in mental health care planning: a qualitative exploration of service user perspectives. J Psychiatr Ment Health Nurs. 2016;23:12‐21. 26634415 10.1111/jpm.12275

[17] Goss C , Moretti F , Mazzi MA , Del Piccolo L , Rimondini M , Zimmerman C . Involving patients in decisions during psychiatric consultations. Br J Psychiatry. 2008;193:416‐421. 18978325 10.1192/bjp.bp.107.048728

[18] Bee P , Brooks H , Fraser C , Lovell K . Professional perspectives on service user and carer involvement in mental health care planning: a qualitative study. Int J Nurs Stud. 2015;52:1834‐1845. 26253574 10.1016/j.ijnurstu.2015.07.008 PMC4642654

[19] Brooks H , Sanders C , Lovell K , Fraser C , Rogers A . Re‐inventing care planning in mental health: stakeholder accounts of the imagined implementation of a user/carer involved intervention. BMC Health Serv Res. 2015;15:490. 26519298 10.1186/s12913-015-1154-z PMC4628327

[20] The Kings Fund . Mental health under pressure. 2015 https://www.kingsfund.org.uk/publications/mental-health-under-pressure. Accessed May 31, 2017.

[21] Lippert ML , Brostrom Kousgaard M , Bjerrum L . General practitioners uses and perceptions of voluntary electronic feedback on treatment outcomes ‐ a qualitative study. BMC Fam Pract. 2014;15:193. 25433487 10.1186/s12875-014-0193-6 PMC4255701

[22] Robert G , Cornwell J . What Matters to Patients? Developing the Evidence Base for Measuring and Improving Patient Experience. London: Kings Fund; 2011.

[23] NHS England . Mental health. https://www.england.nhs.uk/commissioning/spec-services/npc-crg/group-c/. Accessed May 31, 2017.

[24] Lester HE , Hannon KL , Campbell SM . Identifying unintended consequences of quality indicators: a qualitative study. BMJ Qual Saf. 2011;20:1057‐1061. 10.1136/bmjqs.2010.048371 21693464

[25] Bardach NS , Cabana MD . The unintended consequences of quality improvement. Curr Opin Pediatr. 2009;21:777‐782. 19773653 10.1097/MOP.0b013e3283329937 PMC3861327

[26] Rogers A , Vassilev I , Kennedy A . Understanding the dynamics of patient systems of implementation: a mixed methods study. BMC Health Serv Res. 2014;14:O11.

[27] Pilgrim D , Rogers A , Bentall R . The centrality of personal relationships in the creation and amelioration of mental health problems: the current interdisciplinary case. Health. 2009;13:235‐254. 19228830 10.1177/1363459308099686

[28] Tong A , Sainsbury P , Craig J . Consolidated criteria for reporting qualitative research (COREQ): a 32‐item checklist for interviews and focus groups. Int J Qual Health Care. 2007;19:349‐357. 17872937 10.1093/intqhc/mzm042

[29] Furber C . Framework analysis: a method for analysing qualitative data. Afr J Midwifery Womens Health. 2010;4:97‐100.

[30] Richie J , Spencer L . Qualitative data analysis for applied policy research In: Bryman A, Burgess B, eds. Analysing Qualitative Data. London: Routledge; 1994:173‐194.

[31] Pope C , Ziebland S , Mays N . Analysing qualitative data. BMJ. 2000;320:114‐116. 10625273 10.1136/bmj.320.7227.114 PMC1117368

[32] Bower P , Roberts C , O'Leary N , et al. A cluster randomised controlled trial and process evaluation of a training programme for mental health professionals to enhance user involvement in care planning in service users with severe mental health issues (EQUIP): study protocol for a randomised controlled trial. Trials. 2015;16:348. 26268221 10.1186/s13063-015-0896-6 PMC4535374

[33] Coffey M , Cohen RL , Faulkner A , Hannigan B , Simpson A , Barlow S . Ordinary risks and accepted fictions: how contrasting and competing priorities work in risk assessment and mental health care planning. Health Expect. 2017;20:471‐483. 27312732 10.1111/hex.12474 PMC5433531

[34] Quinlivan L , Cooper J , Meehan D , et al. Predictive accuracy of risk scales following self‐hard: multicentre, prospective cohort study. Br J Psychiatry. 2017;210:429‐436. 28302702 10.1192/bjp.bp.116.189993 PMC5451643

[35] Slemon A , Jenkins E , Bungay V . Safety in psychiatric inpatient care: the impact of risk management culture on mental health nursing practice. Nurs Inq. 2017;24:e12199. 10.1111/nin.12199 PMC5655749 28421661

[36] Kennedy A , Vassilev I , James E , Rogers A . Implementing a social network intervention designed to enhance and diversify support for people with long‐term conditions. A qualitative study. Implement Sci. 2016;11:27. 26926837 10.1186/s13012-016-0384-8 PMC4772323

[37] Blakeman T , Blickem C , Kennedy A , et al. Effect of information and telephone‐guided access to community support for people with chronic kidney disease: randomised controlled trial. PLoS One. 2014;9:e109135. 25330169 10.1371/journal.pone.0109135 PMC4199782

[38] Brooks H , Pilgrim D , Rogers A . Innovation in mental health services: what are the key components of success? Implement Sci. 2011;6:120. 22029930 10.1186/1748-5908-6-120 PMC3214129

